# The role of clinically significant portal hypertension in hepatic resection for hepatocellular carcinoma patients: a propensity score matching analysis

**DOI:** 10.1186/s12885-015-1280-3

**Published:** 2015-04-11

**Authors:** Wei He, Qingli Zeng, Yun Zheng, Meixian Chen, Jingxian Shen, Jiliang Qiu, Miao Chen, Ruhai Zou, Yadi Liao, Qijiong Li, Xianqiu Wu, Binkui Li, Yunfei Yuan

**Affiliations:** 1 State Key Laboratory of Oncology in South China and Collaborative Innovation Center for Cancer Medicine, Sun Yat-Sen University Cancer Center, Guangzhou, China; 2 Department of Hepatobiliary Oncology, Sun Yat-Sen University Cancer Center, Guangzhou, China; 3 Department of Medical Imaging and Interventional Center, Sun Yat-Sen University Cancer Center, Guangzhou, China; 4 Department of Surgery, People’s Hospital of Jiangxi Province, Nanchang, China; 5 Department of Ultrasound, Sun Yat-Sen University Cancer Center, Guangzhou, China

**Keywords:** Hepatocellular carcinoma, Hepatic resection, Portal hypertension, Complication, Prognosis

## Abstract

**Background:**

Whether portal hypertension (PHT) is an appropriate contraindication for hepatic resection (HR) in hepatocellular carcinoma (HCC) patient is still under debate.

Aims: Our aim was to assess the impact of clinically significant PHT on postoperative complication and prognosis in HCC patients who undergo HR.

**Methods:**

Two hundred and nine HCC patients who underwent HR as the initial treatment were divided into two groups according to the presence (n = 102) or absence (n = 107) of clinically significant PHT. Propensity score matching (PSM) analysis was used to compare postoperative outcomes and survival.

**Results:**

Before PSM, PHT patients had higher rates of postoperative complication (43.1% *vs*. 23.4%; *P* = 0.002) and liver decompensation (37.3% *vs*. 17.8%; *P* = 0.002) with similar rates of recurrence-free survival (RFS; *P* = 0.369) and overall survival (OS; *P* = 0.205) compared with that of non-PHT patients. However, repeat analysis following PSM revealed similar rates of postoperative complication (32.2% *vs*. 39.0%; *P* = 0.442), liver decompensation (25.4% *vs*. 32.2%; *P* = 0.416), RFS (*P* = 0.481) and OS (*P* = 0.417; 59 patients in each group). Presence of PHT was not associated with complication by logistic regression analysis, or with overall survival by Cox regression analysis.

**Conclusions:**

The presence of clinically significant PHT had no impact on postoperative complication and prognosis, and should not be regarded as a contraindication for HR in HCC patients.

## Background

Liver cancer is the sixth most common cancer, the third cause of cancer-related death and accounts for 7% of all cancers [1]. Curable treatments for hepatocellular carcinoma (HCC) include liver transplantation, hepatic resection (HR) and percutaneous approaches. Portal hypertension (PHT) is considered to be a contraindication for HR in HCC patients according to the 2001 European Association for Study of Liver (EASL). These observations are based on the findings of a study [2] involving 29 patients in 1996 which concluded that PHT, defined as a hepatic venous pressure gradient (HVPG) ≥ 10 mmHg, was a predictor of postoperative liver decompensation [2], and another study in 1999 showing that Child-Pugh A patients with PHT have poor survival after HR [3]. Although this conclusion is supported by subsequent studies [4-7], contradictory reports have also been published indicating that HR is safe and beneficial in well-selected HCC patients with PHT because postoperative outcomes and survival are similar, regardless of the presence or absence of clinically significant of PHT [4,8-11].

Consequently, the role of PHT in the prognosis of HCC patients after HR remains a highly debated topic. Nevertheless, the 2012 EASL-EORTC Clinical Practice Guidelines still advise against HR as the first-line treatment option for HCC patients with PHT, defined as hepatic venous pressure gradient ≥ 10 mmHg or with surrogates of portal hypertension, defined as esophageal varices, or splenomegaly with platelet count < 100,000/mm3 [12].

We therefore conducted a retrospective study involving HCC patients who underwent HR as the initial treatment to assess the role of clinically significant PHT in HCC patients after HR. Propensity score matching was used in the analysis to reduce bias in patient selection and to achieve an even distribution of baseline liver function.

## Methods

### Patients

We retrospectively collected data from 1542 patients who underwent HR as the initial treatment for HCC from January 2003 to December 2008 at Sun Yat-sen University Cancer Center in China. The diagnosis of HCC was confirmed postoperatively by pathological investigation.

HVPG over 10 mmHg was regarded as the gold diagnosis criteria of PHT. It is not, however, routinely used in clinical practice for its invasiveness. Studies show esophageal varices, low platelet count, low white blood cell count [13], splenomegaly, a portal vein diameter on ultrasound (US) ≥13 mm, a high Child-Pugh score, low prothrombin activity, spider angiomas, and a low platelet to spleen ratio are associated with clinically significant PHT [14]. Therefore, portal hypertension was indirectly defined, which incorporated the BCLC criteria [15] and the Italian Programme on Liver Cirrhosis [16, 17] if two or more of the following criteria were met: 1) Platelet count < 100 × 10^9^/l and/or white blood cell count < 4 × 10^9^/l three times in succession, 2) Splenomegaly (spleen thickness > 4.5 cm via ultrasound or major diameter > 10 cm via CT/MR), 3) Portal vein width > 14 mm or spleen vein width > 10 mm via ultrasound, and 4) Esophageal varices via endoscopy or CT/MR.

**Figure 1:**
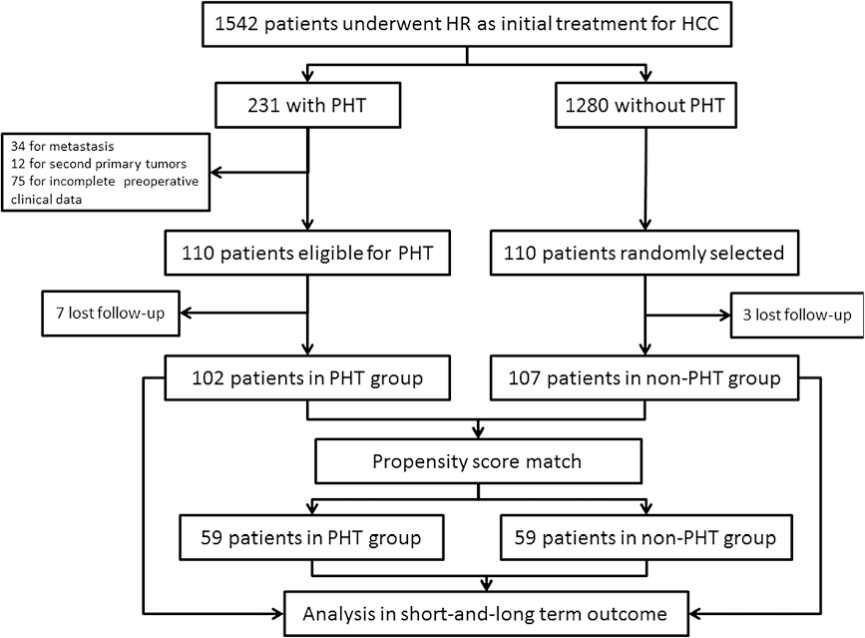
Flow chart.

Of the 1542 patients who underwent HR as initial treatment for HCC, 231 patients were diagnosed with PHT. We subsequently excluded 34 of the 231 PHT patients for hepatic major vessel invasion, 12 for second primary tumors, and 75 for incomplete preoperative clinical data. This left 110 patients in the PHT group eligible for analysis and 110 patients without PHT were randomly selected from the remaining 1280 patients as the control group. There were seven patients in the PHT group and three patients in the non-PHT group lost to follow-up. The study ultimately enrolled 209 patients, with 102 in the PHT group and 107 in the non-PHT group (Figure 1). All the recruited patients provided written informed consent before examination and resection. The study protocol was approved by the Ethics Committee of Sun Yat-Sen University Cancer Center and conformed to the ethical guidelines of the Helsinki Declaration.

### Methods

The hepatic resection strategy was detailed in our previous report [18]. Briefly, we developed a surgical plan according to tumor extent evaluated by preoperative imaging and liver function evaluated by blood biochemistry, Child-Pugh grading, and ICGR15 level. Intraoperative ultrasound was applied to guarantee complete tumor resection and to reduce major vessel injury. A Cavitron ultrasonic surgical aspirator (CUSA) was used to dissect parenchyma. To decrease intraoperative blood loss, we applied the intermittent Pringle maneuver method for the majority of hepatic resections and lowered central venous pressure to 2–4 mmHg routinely. We used blood transfusions to maintain hemoglobin at least 80–90 g/l according to the degree of bleeding and the preoperative hemoglobin level.

Postoperative complications in the first 90 days were recorded and assessed by the Clavien-Dindo classification at grades ranging from one to five, with higher grades indicating greater severity [19]. We defined postoperative liver decompensation as the presence of refractory ascites and edema, hepatic encephalopathy, jaundice with total bilirubin levels above 50 μmol/l [20], renal impairment, and alteration of coagulation factors (INR above 1.50) requiring fresh transfusion. The observation and grading of complications were reviewed independently by two of the authors, and any disagreements were resolved by consensus.

After surgery, all patients were followed up monthly in the first 3 months, every three3 months in the first 2 years and every 3–6 months thereafter. The follow-up strategy was more intensive for patients with recurrence or metastases. Recurrence was observed by measurements of serum alpha-fetoprotein level combined with ultrasound, computed tomography or magnetic resonance imaging. The treatment strategy for recurrence included repeat resection/ablation for patients with three lesions or fewer and transhepatic arterial chemotherapy and embolization (TACE) or sorafenib for patients with more than three lesions. Overall survival was calculated from the time of HR to death or last follow-up until September 2013.

### Statistical analysis

Data were summarized as median for continuous variable and number/prevalence for categorical variable. Continuous variables were compared by the independent samples T-test and Mann–Whitney U test, as appropriate. Binary and ordinal categorical variable were compared by the Chi-square test and Kruskal-Wallis test, respectively. Logistic regression with stepwise manner (entry criterion *P* = 0.05 and removal criterion *P* = 0.1) was used to explore independent prognostic factors of postoperative outcome. Recurrence-free survival (RFS) and overall survival (OS) were assessed by the Kaplan-Meier method using the log-rank test. Cox proportional hazards model in a stepwise manner (entry criterion *P* = 0.05 and removal criterion *P* = 0.1) was used in multivariate analysis to explore independent prognostic factors of overall survival.

Variables with statistically significant differences between groups might have impact on the postoperative outcomes. Therefore we applied propensity score matching (PSM) analysis [21, 22] to minimize the influence of selection bias and potential confounding variables between groups. Potential covariables included in PSM included age, alanine aminotransferase, aspartate aminotransferase, hemoglobin, albumin, total bilirubin, creatinine, prothrombin time, activated partial thromboplastin time, INR, Child-Pugh score, MELD score, extent of hepatectomy, tumor size, and tumor number. PHT related variables were excluded, including white blood cell and platelet count. A one-to-one nearest neighbor matching algorithm was applied with a caliper of 0.2 [23] and without replacement. After matching, 118 patients were included in further analysis (59 in each group). Standardized mean differences were also reported for all variables. Statistical analysis was performed using SPSS (IBM SPSS Statistics for Windows, Version 20.0. IBM Corp., Armonk, NY) and Propensity Score Matching for SPSS, version 1.0 (Felix Thoemmes, Cornell University/University of Tübingen). *P* < 0.05 was considered as statistically significant.

## Results

The baseline characteristics of demographic and clinical variables in both groups are described in Table 1. The most frequent etiology was chronic hepatitis B virus (HBV) in both the PHT and non-PHT groups (93.1% *vs*. 94.4%). In particular, patients in the PHT group have higher total bilirubin levels (19.5 *vs*. 14.6 μmol/l; *P* < 0.001), higher INR values (1.1 *vs*. 1.0; *P* = 0.009), longer prothrombin time (14.2 *vs*. 13.1; *P* < 0.001), longer activated partial thromboplastin time (54.1 *vs*. 35.4; *P* < 0.001), lower creatinine levels (79.7 *vs*. 86.2; *P* = 0.009), lower serum albumin levels (40.7 *vs*. 42.6 g/l; *P* = 0.006), higher Child-Pugh score (5.3 *vs*. 5.1; *P* < 0.001) and higher MELD score (8.8 *vs*. 7.9; *P* < 0.001). Patients with PHT have smaller tumors (5.3 *vs*. 6.5; *P* = 0.006). Both groups have a similar number of tumors (*P* = 0.575), while patients with PHT received less extensive resection.

**Table 1:** **Baseline clinical and intraoperative characteristics of patients before and after PSM**

Variable	Before PSM				After PSM			
	non-PHT (n = 107)	PHT (n = 102)	P	d	non-PHT (n = 59)	PHT (n = 59)	P	d
Age (yr)	48.5 ± 11.4	49.4 ± 11.2	0.468	−0.080	48.7 ± 12.1	49.1 ± 10.8	0.561	−0.035
>65:<65	11:96	12:90	0.732	−0.083	7:46	5:54	0.542	0.274
Gender (male: female)	91:16	85:17	0.734	0.071	51/8	49/10	0.609	0.145
HBV/No-HBV	101/6	95/7	0.707	0.119	55/4	53/6	0.509	0.244
ALB (g/L)	42.6 ± 4.2	40.7 ± 4.9	0.006	0.417	42.1 ± 4.3	42.1 ± 3.9	0.802	−0.001
TBil (μmol/L)	14.6 ± 5.4	19.5 ± 7.3	<0.001	−0.766	16.6 ± 5.6	17.3 ± 5.8	0.611	−0.123
ALT (U/L)	45.2 ± 24.8	50.6 ± 36.5	0.254	−0.174	50.7 ± 28.5	47.2 ± 23.2	0.891	0.135
AST (U/L)	48.5 ± 25.3	51.1 ± 38.2	0.803	−0.081	48.4 ± 19.0	47.9 ± 22.3	0.675	0.024
WBC (10^9^/L)	6.4 ± 1.6	5.2 ± 2.2	<0.001	0.626	6.4 ± 1.8	5.1 ± 2.1	<0.001	0.665
RBC (10^12^/L)	4.6 ± 0.7	4.5 ± 0.7	0.402	0.143	4.5 ± 0.7	4.6 ± 0.8	0.515	−0.133
Hemoglobin (g/L)	138.2 ± 19.5	139.2 ± 19.1	0.776	−0.052	138.3 ± 17.8	141.3 ± 19.2	0.385	−0.162
Thrombocyte (10^9^ /L)	189.4 ± 66.6	96.4 ± 42.4	<0.001	1.657	171.4 ± 63.1	97.9 ± 44.6	<0.001	1.3452
Creatinine (μmol/L)	86.2 ± 19.2	79.7 ± 18.9	0.009	0.341	83.6 ± 19.2	80.7 ± 20.4	0.376	0.146
BUN (mmol/L)	5.1 ± 1.2	7.60 ± 2.6	0.663	−1.245	5.2 ± 1.2	5.1 ± 1.4	0.376	0.077
PT (sec)	13.1 ± 1.2	14.2 ± 1.2	<0.001	−0.917	13.7 ± 0.9	13.8 ± 0.9	0.751	−0.111
APTT (sec)	35.4 ± 5.9	54.1 ± 38.6	<0.001	−0.685	37.1 ± 5.6	37.3 ± 6.2	0.946	−0.034
INR	1.00 ± 0.3	1.1 ± 0.1	<0.001	−0.443	1.1 ± 0.1	1.1 ± 0.1	0.530	0.001
Child-Pugh classification (A/B)	105/2	99/3	0.957	0.256	57/2	59/0	0.476	-
MELD score	7.9 ± 1.4	8.8 ± 1.9	<0.001	−0.541	8.2 ± 1.5	8.3 ± 1.6	0.825	−0.065
MELD classification (<9:9–10:>10)	84:15:8	57:20:25	<0.001	−0.547	42:9:8	41:8:10	0.776	
Size of the tumor (cm)	6.5 ± 3.3	5.3 ± 3.1	0.006	0.375	5.7 ± 3.2	5.5 ± 2.9	0.912	0.066
Vascular invasion(pre: abs)	7:100	15:87	0.055	−0.497	3:56	9:50	0.068	−0.668
Adjacent tissues invasion (pre: abs)	17:90	12:90	0.389	0.192	8:51	9:50	0.793	−0.076
Lymphatic metastasis (pre: abs)	2:105	1:101	1.000	0.361	0	1:58	1.000	-
Number of tumor (1:2:>2)	77:15:15	71:11:20	0.575	−0.104	42:7:10	41:8:10	0.870	−0.022
Extent of hepatectomy (1:2:>2)	46:36:25	55:36:11	0.034	0.319	29:20:10	35:16:8	0.289	0.185
Intraoperative heamorrhage > 400 ml	25	28	0.497	−0.119	8	16	0.067	−0.476
Intraoperative transfusion	20	24	0.391	−0.161	6	11	0.190	−0.389
Pringle maneuver	85	76	0.397	0.154	50	46	0.344	0.249

Continuous variables are reported in mean and standard deviation.

d indicates standardized mean differences.

As shown in Table 2, patients in the PHT group have higher liver decompensation rates (37.3% *vs*. 17.8%; *P* = 0.002), higher postoperative complication rate (43.1% *vs*. 23.4%; *P* = 0.002) and stayed longer in hospital (12.9 days *vs*.12.1 days; *P* < 0.001), compared with those of the non-PHT group. However, the distribution of severity of postoperative complication in both groups was similar (*P* = 0.818); seven deaths occurred within 90 days as a result of liver failure, five in the PHT group and two in the non-PHT group. The mortality rate was similar (4.90% *vs*. 1.87%; *P* = 0.271).

**Table 2:** **Postoperative outcomes before and after PSM**

Variables	Before PSM			After PSM		
	non-PHT (n = 107)	PHT (n = 102)	P	non-PHT (n = 59)	PHT (n = 59)	P
Hospital stay (days)	12.1 ± 5.3	12.9 ± 3.9	<0.001	12.6 ± 6.1	12.8 ± 4.2	0.082
Liver decompensation	19(17.8%)	38(37.3%)	0.002	15(25.4%)	19(32.2%)	0.416
Complication	25(23.4%)	44(43.1%)	0.002	19(32.2%)	23(39.0%)	0.442
Clavien-Dindo Classification			0.818			0.929
Grade 1	17(15.7%)	31(30.4%)		12(20.3%)	15(25.4%)	
Grade 2	2(1.9)	5(4.9%)		2(3.4%)	3(5.1%)	
Grade 3	3(2.8%)	3(2.9%)		3(5.1%)	2(3.4%)	
Grade 4	1(0.9%)	0		1(1.7%)	0	
Grade 5	2(1.9%)	5(4.9%)		1(1.7%)	3(5.1%)	

Univariate analysis (Table 3) shows that postoperative complications occurred more frequently in patients with PHT (*P* = 0.003), age > 65 (*P* = 0.043), requiring intraoperative transfusion (*P* = 0.021), INR > 1.20 (*P* = 0.044) and with a greater number of tumors (*P* = 0.031). Liver decompensation occurred more frequently in patients with PHT (*P* = 0.002), intraoperative hemorrhage > 400 ml (*P* = 0.050), requiring intraoperative transfusion (*P* = 0.003), INR > 1.2 (*P* = 0.017) and with a greater number of tumors (*P* = 0.018).

**Table 3:** **Univariate analysis of predictive factors of postoperative complication and liver decompensation**

Variables	Postoperative complication				Liver decompensation			
	Before PSM		After PSM		Before PSM		After PSM	
	Prevalence	P	Prevalence	P	Prevalence	P	Prevalence	P
**Age > 65y (Yes : No)**	52.2% : 30.6%	0.043	50.0% : 34.0%	0.278	34.8% : 26.3%	0.394	25.0% : 29.2%	0.759
**Gender (Male : Female)**	31.8% : 39.4%	0.397	35.6% : 38.9%	0.751	27.8% : 24.2%	0.670	31.0% : 16.7%	0.226
**Etiology of cirrhosis (HBV : Non-HBV)**	32.7% : 38.5%	0.667	34.3% : 50.0%	0.326	28.1% : 15.4%	0.331	29.6% : 20.0%	0.524
**ALB (<35 : >35)**	47.1% : 31.8%	0.205	50.0% : 34.8%	0.456	41.2% : 26.0%	0.186	33.3% : 28.6%	0.802
**TBIL (<34.2 : >34.2)**	33.3% : 0	0.999	35.6% : 0	0.999	27.5% : 0	0.999	0 : 0	-
**INR (<1.2 : >1.2)**	30.4% : 50.0%	0.044	34.3% : 50.0%	0.326	24.3% : 46.4%	0.017	26.9% : 50.0%	0.134
**Child-Pugh classification (A : B)**	32.4% : 60.0%	0.217	35.3%: 50.0%	0.672	40.0% : 27.0%	0.523	0 : 29.3%	0.999
**MELD (<9 : 9–10 : >10)**	31.2% : 42.9% : 30.3%	0.746	36.1% : 47.1% : 22.2%	0.460	24.1% : 40.0% : 27.3%	0.359	27.7% : 41.2% : 22.2%	0.941
**Size of tumor (<5 : >5)**	33.9% : 32.0%	0.763	30.0% : 43.8%	0.127	25.0% : 29.9%	0.428	20.0% : 41.7%	0.012
**Vascular invasion (Yes : No)**	36.4% : 32.6%	0.724	42.9% : 34.6%	0.547	31.8% : 26.7%	0.613	35.7% : 27.9%	0.545
**Adjacent tissues invasion (Yes : No)**	31.0% : 33.3%	0.807	35.3% : 35.6%	0.978	31.0% : 26.7%	0.624	35.3% : 27.7%	0.525
**Lymphatic metastasis (Yes : No)**	0 : 33.5%	0.999	0 : 35.9%	1.000	0 : 27.7%	0.999	0% : 29.1%	1.000
**Number of tumor (1 : 2 : >2)**	28.4% : 42.3% : 45.7%	0.031	30.1% : 53.3% : 45.0%	0.111	23.0% : 30.8% : 42.9%	0.018	25.3% : 33.3% : 40.0%	0.176
**PHT (Yes : No)**	43.1% : 23.4%	0.003	39.0% : 32.2%	0.442	37.3% : 17.8%	0.002	32.2% : 25.4%	0.417
**Extent of hepatectomy (1 : 2 : >2)**	28.7% : 40.3% : 30.6%	0.496	28.1% : 44.4% : 44.4%	0.099	23.8% : 31.9% : 27.8%	0.439	21.9% : 36.1% : 38.9%	0.088
**Intraoperative hemorrhage >400 ml (Yes : No)**	41.5% : 30.1%	0.130	54.2% : 30.9%	0.037	37.7% : 23.7%	0.050	50.0% : 23.4%	0.013
**Intraoperative Transfusion (Yes : No)**	47.7% : 29.1%	0.021	58.8% : 31.7%	0.036	45.5% : 22.4%	0.003	52.9% : 24.8%	0.022
**Pringle maneuver (Yes : No)**	33.5% : 31.2%	0.767	35.4% : 36.4%	0.933	28.0% : 25.0%	0.687	29.2% : 27.3%	0.860

Multivariate analysis showed that the presence of PHT (Odds ratio: 2.415; 95% Confidence interval: 1.307–4.463; *P* = 0.050), age > 65 (OR: 2.683; 95%CI: 1.069–6.733; *P* = 0.036) and multiple tumor number (OR: 1.494; 95%CI: 1.013–2.203; *P* = 0.043) were significant predictors for postoperative complication. The presence of PHT (OR: 2.650; 95%CI: 1.375–5.110; *P* = 0.004), intraoperative transfusion (OR: 2.687; 95%CI: 1.293–5.584; *P* = 0.008) and greater number of tumors (OR: 1.495; 95% CI: 1.003–2.229; *P* = 0.048) were significant predictors for liver decompensation. Both groups had similar RFS rates (*P* = 0.369) with a median recurrence-free time of 23.57 months and similar OS rates (*P* = 0.205) with a median follow-up of 56.60 months. The OS rates of 1, 3 and 5 years were 82%, 59%, 46% in the PHT group and 86%, 65%, 50% in the non-PHT group, respectively. After recurrence, 19 patients underwent resection, 36 patients underwent percutaneous microwave coagulation or radiofrequency ablation, 62 patients received TACE and four patients received sorafenib treatment.

### Results after propensity score matching

**Figure 2:**
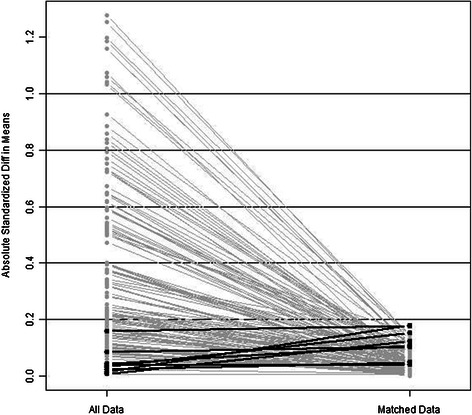
Parallel line plot of the standardized difference in means before and after PSM in HCC patients with and without PHT. As the standardized difference in means was reduced, covariate balance was improved in the matched samples.

**Figure 3:**
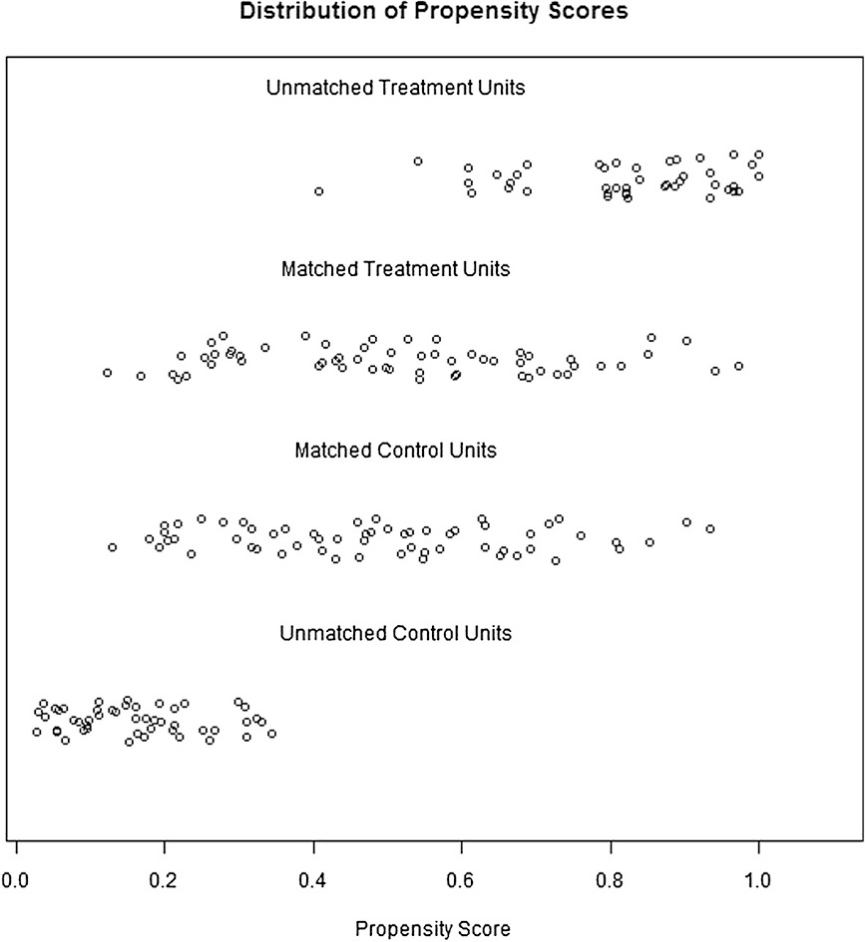
Dot plot of the propensity scores of patients in the PHT and the non-PHT groups showing individual units in the dataset and whether they were matched or discarded.

The demographic and clinical characteristics were generally similar in both groups (59 in the PHT group and 59 in the non-PHT group) after covariates had been adjusted by PSM (Table 1). The standardized difference in means and individual propensity scores of patients is shown in Figures 2 and 3. Variables that were unevenly distributed before PMS included age > 65 (*P* = 0.542), INR (*P* = 0.530), number of tumors (*P* = 0.870) and intraoperative transfusion (*P* = 0.067).

The postoperative outcome after PSM matching is shown in Table 2. No significant difference in outcome was found between the two groups. Specifically, both groups had a similar length of hospital stay (12.64 *vs*. 12.83; *P* = 0.082), liver decompensation rate (25.4% *vs*. 32.2%; *P* = 0.416), postoperative complication rate (32.2% *vs*. 39.0%; *P* = 0.442) and distribution of severity of complication (*P* = 0.929). Four death events occurred within 90 days because of postoperative liver failure, three in the PHT group and one in the non-PHT group with similar mortality rate (5.08% *vs*. 1.69%; *P* = 0.619).

Univariate analysis in matched patients showed that postoperative complication and liver decompensation occurred more frequently in patients with intraoperative hemorrhage more than 400 ml (*P* = 0.037 and *P* = 0.013, respectively) and requiring intraoperative transfusion (*P* = 0.036 and *P* = 0.022, respectively; Table 3).

**Figure 4:**
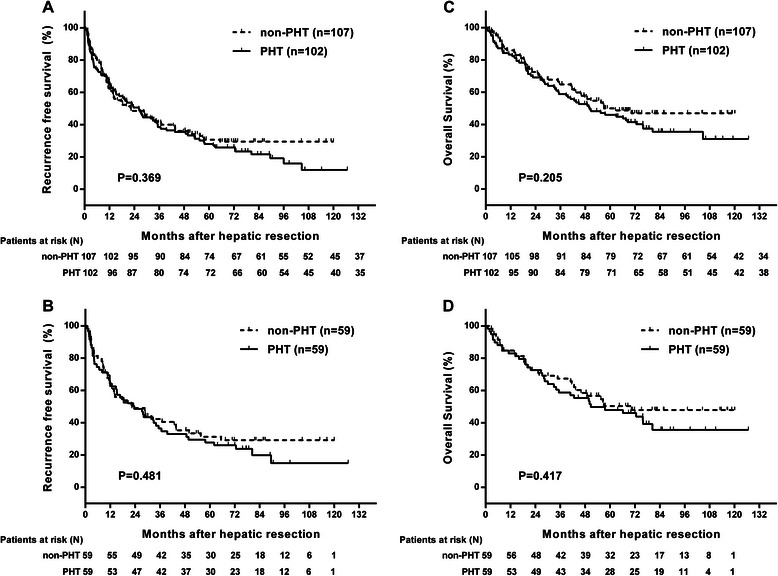
Kaplan-Meier survival curves in HCC patients who underwent HR. **(A)** and **(B)** show recurrence-free survival in the PHT and non-PHT groups before and after PSM, respectively. **(C)** and **(D)** show overall survival between the PHT and the non-PHT groups before and after PSM, respectively.

Logistic regression identified intraoperative transfusion (OR: 3.080; 95%CI: 1.075–8.828; *P* = 0.036) and intraoperative hemorrhage of more than 400 ml (OR: 3.273; 95%CI: 1.289–8.310; *P* = 0.013) as independent predictive factors of postoperative complication and liver decompensation, respectively. Both groups had similar RFS rates (*P* = 0.481) with a median RFS time of 23.07 months and similar OS rates (*P* = 0.417) with a median follow-up of 57.57 months. OS rates of 1, 3, and 5 years are 83%, 59%, 48% in the PHT group and 85%, 67%, 50% in the non-PHT group (Figure 4). Multivariate Cox proportional hazard regression (Table 4) identified MELD score (RR: 2.113; 95%CI: 1.242-3.593, P = 0.006), number of tumors (RR: 2.020, 95%CI: 1.192-3.424, P = 0.009), vascular invasion (RR: 2.289, 95%CI: 1.137-4.612, P = 0.020), adjacent tissues invasion (RR: 2.549, 95%CI: 1.372-4.735, P = 0.003) and intraoperative hemorrhage > 400 ml (RR: 1.849, 95%CI: 1.027-3.330, P = 0.041) as predictors for death. Importantly, PHT was not identified as a predictor for death in the post-PSM Cox regression analysis.

**Table 4:** **Univariate and multivariate analysis of predictive factors of overall survival after PSM**

Variables	Univariate analysis		Multivariate analysis		
	χ2	P	RR	95% CI	P
Age > 65y (Yes : No)	0.001	0.982			
Gender (Male : Female)	1.945	0.163			
Etiology of cirrhosis (HBV: non-HBV)	0.001	0.976			
ALB (<35 : >35)	0.018	0.892			
INR (<1.2 : >1.2)	2.930	0.087			
Child-Pugh classification (A : B)	1.994	0.158			
MELD (<9 : >9)	3.480	0.065	2.113	1.242-3.593	0.006
PHT (Yes : No)	0.660	0.417			
Number of tumor (1 : >1)	10.134	0.001	2.020	1.192-3.424	0.009
Size of tumor (<5 : >5)	5.604	0.018			
Vascular invasion (Yes : No)	13.767	<0.001	2.289	1.137-4.612	0.020
Adjacent tissues invasion (Yes : No)	11.552	0.001	2.549	1.372-4.735	0.003
Intraoperative haemorrhage >400 ml (Yes : No)	8.770	0.003	1.849	1.027-3.330	0.041
Extent of hepatectomy (1 : >1)	0.443	0.506			
Intraoperative Transfusion (Yes : No)	2.565	0.109			
Pringle maneuver (Yes : No)	0.287	0.592			

Lymphatic metastasis is not included in analysis because there is only 1 case in 118 patients after matching.

### Characteristics and outcome of unmatched patients

Forty-eight patients were excluded from the non-PHT group by PSM. These patients had a mean tumor size of 7.29 ± 3.33 cm, a CTP score of 5.04 ± 0.20, and a MELD score of 7.54 ± 1.14. There were five grade I complications, no grade II, III or IV complications, and one grade V complication, and the 1, 3, and 5 year survival rates were 88%, 63% and 49%, respectively. Forty-three patients were excluded from the PHT group by PSM. The excluded patients had a mean tumor size of 5.20 ± 3.48 cm, a CTP score of 5.54 ± 0.71 and a MELD score of 9.61 ± 1.97. Grade I, II, III, IV, and V complications occurred in 16, 2, 1, 0 and 2 cases, respectively, and the 1-, 3- and 5-year survival rates were 80%, 57% and 43%, respectively.

## Discussion

In 2001, the EASL (European Association for Study of Liver) concluded that PHT was a contraindication for HR [24] based on a 29-case cohort in which HCC patients had higher risk of hepatic decompensation(73.3% *vs*. 0%,P < 0.0002)when PHT was diagnosed by HVPG ≥ 10 mmHg [2]. Further evidence of the impact of PHT on long-term survival, was taken from a later study which found that HVPG ≥ 10 mmHg or clinically significant PHT was an independent prognostic factor [3]. These results are supported by multiple regional studies over the past decade suggesting that HCC patients indirectly diagnosed with PHT [5-7,11] or directly diagnosed by HVPG [25,26] more frequently suffer from severe postoperative hepatic decompensation and have shorter survival. Although HVPG has a robust prognostic value [25,26], it is not routinely applied in practice because of invasiveness, high cost and patient discomfort. As a result, the EASL-EORTC clinical practice guidelines also suggest surrogate measurements for the diagnosis of portal hypertension, including platelet count below 100,000/mm^3^ associated with splenomegaly and oesophageal varices [12].

However, several other large studies [4,8-11] reported no significant difference in postoperative outcomes between PHT and non-PHT HCC patients when PHT is diagnosed with these clinical criteria. In Child-Pugh class A patients, no difference was found in postoperative mortality, morbidity [8] and survival [27] between the PHT and the non-PHT group. Similarly Cucchetti et al. reported that portal hypertension had no effect on postoperative outcome and survival as long as other prognostic variables were evenly distributed between both groups [4]. Additionally, Child-Pugh A or B patients with esophageal varices have a significantly higher 5-year overall survival rate and a similar postoperative complication rate when compared with patients without esophageal varices [9]. A further study of 434 patients found that PHT was not a prognostic factor with regard to the overall survival, and concluded that resection for HCC may also be applied to patients with PHT [10]. These reports, therefore, strongly suggest that if the liver function of HCC patients is well-preserved, postoperative outcome and survival is similar regardless of presence or absence of clinically significant PHT.

In this retrospective study, the presence of clinically significant PHT was not found to be a predictor of postoperative complication or liver decompensation in univariate and multivariate analysis when other variables were evenly distributed by PSM. The inferior postoperative outcome of PHT patients before matching may be explained by poor preoperative liver function, which may exaggerate the impact of PHT on HCC patients. After matching, both groups show similar demographic and clinical characteristics, suggesting that PSM efficiently eliminates covariates and minimizes their confounding effect, thus resulting in the similar intraoperative and postoperative outcomes. Specifically, there was no difference in the frequency or distribution of severity of postoperative outcomes. The only prognostic variables affecting the postoperative complication and liver decompensation rates were intraoperative transfusion and intraoperative hemorrhage of more than 400 ml, respectively. This observation is supported by previous studies [28-30]. HCC patients with cirrhosis are more likely to have coagulopathy, leading to a higher risk of hemorrhage and intraoperative transfusion requirement. The similar RFS and OS in both groups before and after PSM are consistent with results in previous studies [4,9,31], indicating that HCC patients may still benefit from HR regardless of presence of PHT. This result is also confirmed by multivariate analysis indicating that only lower MELD score, an advanced stage of tumor and ineffective management of intraoperative hemorrhage were prognostic factors for overall survival. With the refinement of patient selection and improvement in surgical management, especially in management of intraoperative hemorrhage, HCC patients with clinically diagnosed PHT can achieve acceptable postoperative outcome.

Although the 2012 EASL-EORTC Clinical Practice Guidelines [12] mention that portal hypertension is a valid prognostic factor in patients undergoing resection in Asia [10], most cirrhotic patients in these studies have an etiological background of hepatitis C virus (HCV) and alcohol, rather than HBV (Table 5). In contrast, HBV is the largest attributable factor (60%) for HCC [32] in Africa and East Asia, while in Europe 60–70% of cases can be attributed to the HCV infection [33]. Consideration should therefore be given to etiological differences in these regions and their influence on postoperative outcome should be evaluated. Importantly, post-resection mortality has been reported to be significantly higher in alcoholic and HCV patients than in HBV patients [34], and an improved rate of OS has been observed in HBV HCC patients compared with those with HCV [35]. Indeed, to date, few studies have been conducted in HBV prevalent regions that support recommendations of the guidelines on this issue [6, 7], with one of these pointing out that even if PHT is a powerful prognostic factor, patients with a single tumor and absence of gross vascular invasion may still experience a survival benefit from HR [7]. We therefore surmise that the recommendation made in the guidelines that PHT should be a contraindication for HR is not appropriate for all patients in these regions. The current study, to the best of our knowledge, is the first study demonstrating that clinically significant PHT is neither a prognostic factor for postoperative complication nor for overall survival after HR in a hepatitis B virus prevalent region.

**Table 5:** **Review of etiological characteristics of patient cohorts in previous studies of PHT**

Study (year)	Patients	Etiology			
		HBV	HCV	Alcohol	Others*
Nagasue et al. (1999) [36]	63	16 (25.40%)	29 (46.03%)	—	18 (28.57%)
Llovet, J. M. (1999) [3]	164	17 (10.37%)	122 (74.39%)	16 (9.76%)	9 (5.49%)
Ripoll, C. (2005) [37]	393	35(8.9%)	142(36.1%)	172(43.8%)	44 (11.20%)
An M (2006) [6]	142	115 (80.99%)	6 (4.23%)	—	21 (14.79%)
Capussotti, L. (2006) [8]	217	43 (19.82%)	105 (48.39%)	62 (28.57%)	7 (3.23%)
Minagawa, M. (2007) [5]	13566	2682 (19.77%)	9025 (66.53%)	3063 (22.58%)	—
Kawano, Y. (2008) [9]	134	29 (21.64%)	75 (55.97%)	—	30 (22.39%)
Ishizawa T (2008) [10]	386	75 (19.43%)	251 (65.03%)	—	
Cucchetti, A. (2009) [4]	241	37 (15.35%)	162 (67.22%)	—	42 (17.43%)
Choi, G. H. (2011) [7]	100	77 (77%)	—	—	—
Boleslawski, E. (2012) [25]	40	5 (12.5%)	30 (75%)	5 (12.5%)	
Santambrogio, R. (2013) [11]	223	38 (17.04%)	135 (60.54%)	—	50 (22.41%)

*Others etiologies include HBV + HCV, hemochromatosis, metabolic, cholestasis and unknown factors.

Nevertheless, our study has several limitations. All patient data were obtained from a single institution. Thus, multiple-center studies will be necessary to validate our conclusions. In addition, PHT in this retrospective study was indirectly diagnosed by clinical criteria so we are still not able to address the role of HVPG measurement as the diagnostic criterion for PHT in HCC patients with HBV cirrhosis.

## Conclusion

In conclusion, with respect to postoperative complication, liver decompensation, RFS and OS, clinically significant PHT should not be regarded as contraindication for HR because HCC patients in HBV prevalent region can still benefit from surgery if preoperative liver function is well-preserved and intraoperative hemorrhage is under control.

## Abbreviations

HCC: Hepatocellular carcinoma)
PHT: Portal hypertension
PSM: Propensity score matching
HBV: Hepatitis B virus
HCV: Hepatitis C virus
ALB: Albumin
TBIL: Total bilirubin
ALT: Alanine aminotransferase
AST: Aspartate aminotransferase
WBC: White blood cell
RBC: Red blood cell
BUN: Blood urea nitrogen
PT: Prothrombin time
APTT: Activated partial thromboplastin time)
INR: International normalized ratio
MELD: Model for end stage liver disease
